# Hematopoietic stem cell transplantation in an infant with dedicator of cytokinesis 8 (DOCK8) deficiency associated with systemic lupus erythematosus: A case report

**DOI:** 10.1097/MD.0000000000020866

**Published:** 2021-04-02

**Authors:** Euri Seo, Beom Hee Lee, Joo Hoon Lee, Young Seo Park, Ho Joon Im, Jina Lee

**Affiliations:** a Department of Pediatrics, Asan Medical Center Children's Hospital, University of Ulsan College of Medicine, Seoul; b Department of Pediatrics, Dongguk University Ilsan Hospital, Goyang, Korea.

**Keywords:** DOCK8 deficiency, SLE, children, HSCT

## Abstract

**Introduction:**

DOCK8 deficiency is a primary immunodeficiency characterized by recurrent infections, severe allergic disease, and autoimmunity. Here, we report a patient with DOCK8 deficiency that was initially presented as systemic lupus erythematosus (SLE) without recurrent infections and treated with hematopoietic stem cell transplantation (HSCT).

**Patient concerns:**

A 16-month-old boy with a previous history of eczema developed high fever and hand and foot swelling. Over time, multiple purpura, oral ulcers, and oliguria developed with a persistent fever. His laboratory findings showed anemia, thrombocytopenia, and coagulopathy with a high level of C-reactive protein (CRP). No definite pathogens were identified. The complement fractions C3, C4, and CH50 were low. Autoantibodies including antinuclear antibody (ANA) and anti-ds DNA antibody were positive. He definitively satisfied the 2015 ACR/SLICC revised criteria for the diagnosis of SLE (7 points out of 16); therefore, he was treated with a steroid. Lupus nephritis was confirmed by renal biopsy later. Considering the early-onset SLE, partial exome sequencing was performed.

**Diagnosis:**

One heterozygous missense variant, c.5536A>G (p.Lys1846Glu), which was inherited from his father, and heterozygous deletion of exon 1 to 8 inherited from his mother were found. Through the results of the genetic testing, the patient was confirmed to have DOCK8 deficiency.

**Interventions:**

At the age of 28 months, he received haploidentical HSCT from his mother as a donor.

**Outcomes:**

Laboratory findings including complement fractions C3, C4, CH50, anti-ds DNA antibody, and the ANA became normal after HSCT. Currently, at 12 months post-HSCT, he is doing well, without any autoimmune features or infections.

**Conclusions:**

DOCK8 deficiency can be presented as autoimmune disease such as SLE. Encountering a child diagnosed with SLE at a very young age, pediatricians should consider immunodeficiency syndrome including DOCK8 deficiency.

## 1 Introduction

Dedicator of cytokinesis 8 protein (DOCK8) deficiency is a primary immunodeficiency disease caused by loss-of-function mutations in the *DOCK8* gene, which is known to play a critical role in the proliferation, migration, survival, and function of several cell types in the immune system, especially lymphocytes.^[1]^ DOCK8 deficiency is the most common cause of autosomal recessive hyper-immunoglobulin E syndrome (HIES) and is mainly expressed as recurrent infections and severe allergic diseases affecting the skin.^[2]^ In addition, autoimmune features, including systemic lupus erythematosus (SLE), vasculitis, and autoimmune hemolytic anemia may be presented in DOCK8 deficiency.^[2–5]^

Autoimmune characteristics in DOCK8 deficiency result from the impairment of regulatory T cells (Tregs) that maintain self-tolerance by suppressing autoreactive B cells.^[6]^ SLE is one of the autoimmune disorders and results from the loss of immunological tolerance. Therefore, defective Tregs are possible pathogenic mechanisms of SLE.^[7]^ DOCK8 deficiency might overlap with these autoimmune characteristics of SLE via defects in Tregs function.

Here, we report a case of a 16-month-old boy diagnosed with DOCK8 deficiency that was initially presented as SLE without recurrent infections. Informed written consent was obtained from his parents for publication of this case report.

## 2 Case presentation

A 16-month-old boy was transferred from an outside hospital because of a high fever and skin rash. He was born via normal vaginal delivery at 40 weeks of gestation, with a birth weight of 3670 g. He was previously healthy except for atopic dermatitis and a history of suspected viral meningitis at the age of 100 days, which was resolved without complication. Age-appropriate vaccination was done without specific adverse events following the administration of the vaccines, which included the Bacillus Calmette–Guérin (BCG), measles/mumps/rubella, and varicella vaccines. His language and motor development were within the normal ranges. On the day of admission, he had a weight of 11.5 kg (50th–75th percentile) and a height of 81.9 cm (50th–75th percentile).

He was admitted to the outside hospital because of a high fever of up to 40°C for 3 days. On physical examination, abnormal findings were not shown other than hand and foot swelling. The initial complete blood count (CBC), blood urea nitrogen (BUN), creatinine (Cr), and serum albumin levels were normal. However, his C-reactive protein (CRP) level was high, at 10.37 mg/dL (the normal level is < 0.5 mg/dL). He was treated with an initial empiric antibiotic of intravenous cefotaxime. On his third day of hospitalization, perioral ecchymosis and mild coagulopathy developed, with a prothrombin time (PT) international normalized ratio (INR) of 1.30, activated partial thromboplastin time (aPTT) of 67.0 seconds, and D-dimer of > 1000 ng/mL. No bacterial or viral pathogens were identified in the initial blood culture and respiratory viral polymerase chain reaction (PCR), respectively. Besides, oliguria (urination of 0.5 cc/kg/h) developed, along with persistent high fever and swollen extremities. He was transferred to our hospital on the sixth day of fever.

**Figure 1:**
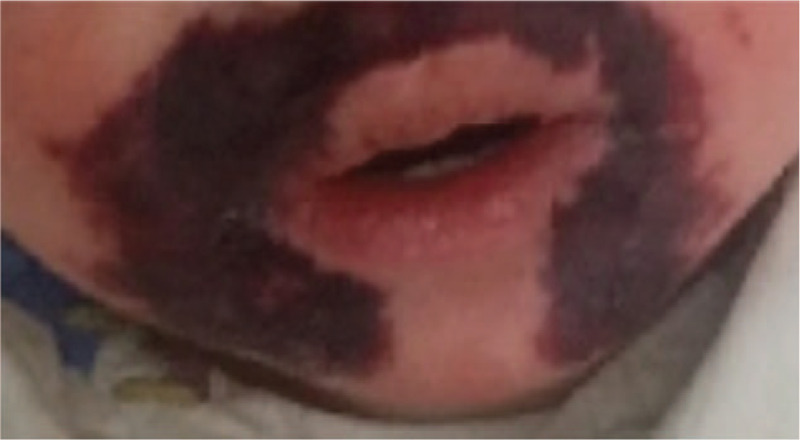
Perioral ecchymosis presentation upon patient's arrival to our hospital.

On the first day at our hospital, his vital signs were as follows: a blood pressure of 149/91 mm Hg (reference: 86–103/40–55); a pulse of 189 bpm (reference: 70–160); and a body temperature of 38.2°C. He appeared acutely ill. Generalized edema, oral ulcers, and multiple purpura, including perioral ecchymosis, were observed on physical examination (Fig. 1). His blood count showed bicytopenia with hemoglobin of 9.5 g/dL, platelet count of 120,000/μL, and white blood cell (WBC) count of 7200/μL, of which 70.7% were neutrophils, 15.8% were lymphocytes, and 4.8% were eosinophils. Other blood laboratory findings were as follows: CRP of 11.14 mg/dL, BUN/Cr of 3 mg/dL / 0.17 mg/dL, albumin of 2.3 mg/dL, PT INR of 1.22, aPTT of 47.8 seconds, fibrinogen of 372 mg/dL, D-dimer of 5.82 μg/mL, and fibrin degradation product of 33 μg/mL. The cerebrospinal fluid profile and urinalysis were unremarkable. Under the impression of serious bacterial infection, he was given vancomycin (60 mg/kg/day, intravenous) and meropenem (60 mg/kg/day, intravenous) as empiric antibiotics. However, repeated culture studies showed no definite pathogens. On the third day, new ecchymosis around his right ear and forehead developed, and a high spiking fever persisted.

To exclude other etiology of fever of an unknown origin, a peripheral blood smear, bone marrow examination, and bone scan were done, which showed no specific abnormalities suggesting malignancies. The immunoglobulin (Ig) G, M, and A levels were normal [at 969 mg/dL (normal: 345–1236), 242 mg/dL (normal: 43–207), and 74.3 mg/dL (normal: 14–159), respectively]. However, the immunoglobulin E (IgE) level was high, at 1679 kU/L (normal: 0–230), and the complement fractions C3, C4, and CH50 were low, at 80.0 mg/dL (normal: 77–195), 6.7 mg/dL (normal: 7–40), and 13.3 U/mL, respectively. The antinuclear antibody (ANA) was positive, at 1:320 with homogenous fluorescence, and the anti-double stranded (anti-ds) DNA antibody was also positive, at 10.8 IU/mL. The tests for anti-SSA, anti-SSB, anti-ribonucleoprotein, anti-scleroderma 70, and anti Jo1 antibodies were all negative.

Two weeks after he was hospitalized, leukopenia developed, and thrombocytopenia became worse, along with the persistent fever. The complement fractions were decreased to 37.9 mg/dL of C3, < 1.5 mg/dL of C4, and under 10.0 U/mL of CH50, respectively. The ANA titer increased to 1:1280 with homogenous fluorescence, and the anti-ds DNA antibody level increased to 84.4 IU/mL. The lupus anticoagulant and anticardiolipin antibody were all positive, while other autoantibodies were all negative. To control the fever, he was started on naproxen on the 30th day of fever, and his fever subsided from 32nd day of fever. He definitively satisfied the 2015 American College of Rheumatology (ACR)/Systemic Lupus Erythematosus International Collaborating Clinics (SLICC) revised criteria for the diagnosis of SLE (7 points out of 16); therefore, he was treated with a steroid (methylprednisolone, 2 mg/kg/day, intravenous). The anti-ds DNA antibody level and complement fractions were improved after steroid therapy, and his fever did not recur.

Approximately 1 week after starting steroid treatment, his steroid dose was reduced to 1.0 mg/kg/day, orally. Two weeks after the first dose reduction, his steroid dose was again reduced to 0.5 mg/kg/day. However, at this point, his fever returned, and his anti-ds DNA antibody titer increased. Accordingly, his steroid dose was increased to 1.0 mg/kg/day, and his fever disappeared. One week later, fever, the swelling of the hand and foot, and proteinuria (Urine Protein/Creatinine ratio of 0.484 mg/mg) appeared again. A renal biopsy confirmed the diagnosis of lupus nephritis (focal lupus nephritis, class III). Transthoracic echocardiography showed vegetation in the tricuspid valve, which was thought to be indicative of Libman-Sacks endocarditis. He was treated with intravenous methylprednisolone (1.0 g/m^2^) for 3 days, followed by cyclophosphamide (500 mg/m^2^). Then, steroid (deflazacort), mycophenolate mofetil, and cyclosporin were used for his SLE, and the disease was well controlled.

Considering the early-onset SLE, partial exome sequencing for 4813 OMIM genes was performed in his peripheral leukocytes. Exomes were captured using the TruSight One Panel (Illumina Inc.), which enriches a 12 Mb region spanning 4813 genes. Sequencing was performed using the NextSeq platform (Illumina Inc.). Sequence reads were aligned to the reference genome, hg19, using a Burrows–Wheeler aligner (BWA) (v0.7.12, MEM algorithm). The mean depth of coverage was 90× (> 10× = 98%). One heterozygous missense variant, c.5536A>G (p.Lys1846Glu), was found, which was inherited from his father. This variant was not reported previously. The mutation was confirmed using in silico prediction tools, such as PolyPhen-2 (http://genetics.bwh.harvard.edu/pph2, score 1.00) and SIFT (http://sift.jcvi.org, score 0.00). This variant has been classified as likely to be pathogenic, according to the American College of Medical Genetics and Genomics (ACMG).^[8]^ To rule out the possibility of a large exonic deletion of DOCK8, multiple ligation-dependent probe amplification (MLPA) analysis (P385-A2, MRC Holland, Amsterdam, the Netherlands) was done in the peripheral leukocytes of both the patient and his parents, which revealed a heterozygous deletion of exon 1 to 8 in the patient and his mother. Through the results of the genetic testing, he was confirmed to have DOCK8 deficiency.

At the age of 22 months, when the patient's anti-ds DNA antibody titer and complement levels were normal as a result of the use of immunosuppressants, he newly experienced skin and soft tissue infections and bacteremia caused by *Pseudomonas aeruginosa* and received cefepime (60 mg/kg/day, intravenous). From that point, he experienced skin and soft tissue infections several times, which is compatible with ecthyma gangrenosum.

**Figure 2:**
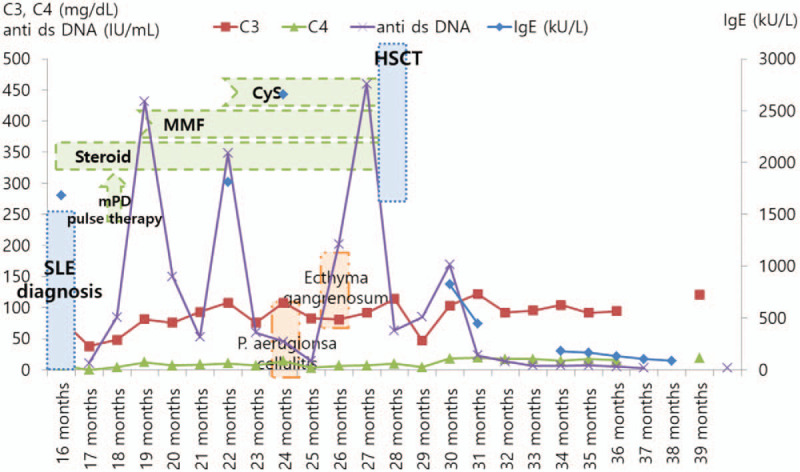
Clinical course of therapeutic drugs and symptoms associated with hyper IgE syndrome; hematopoietic stem cell transplantation resulted in the resolution of symptoms and laboratory findings of systemic lupus erythematosus and hyperimmunoglobulin E syndrome.

At the age of 28 months, he received haploidentical hematopoietic stem cell transplantation (HSCT) from his mother as a donor, with a non-myeloablative conditioning regimen that included antithymocyte globulin, thiosulfan, and fludarabine. WBCs were grafted on the 10th day following HSCT, and no significant HSCT-related morbidity, including graft versus host disease (GvHD), occurred during a follow-up period of 1-year post-HSCT. At 1 month post-HSCT, the complement fractions C3, C4, and CH50 were normal, at 113.0 mg/dL, 22.7 mg/dL, and 51.2 U/mL, respectively. The ANA was positive at 1:160, with homogenous fluorescence, and the anti-ds DNA antibody level had decreased to 13.0. The IgE level was high, at 526 kU/L. However, the anti-ds DNA antibody normalized at 3 months post-HSCT, and the ANA and IgE became normal at 6 months post-HSCT. He had 20% and 33% donor T-cell chimerism at 3 months and 6 months post-HSCT, respectively. Currently, at 12 months post-HSCT, he is doing well, without any autoimmune features or recurrent infections (Fig. 2).

## 3 Discussion

The *DOCK8* gene, which is located at chromosome 9p24.3, encodes a member of the DOCK family of proteins.^[9]^ DOCK family proteins are involved in chemical signaling within cells, and this signaling affects the arrangement of the cytoskeleton. By controlling the structure of the cells, DOCK family proteins help cells maintain their structure and move to their target site. DOCK8 protein is most abundant in immune system cells, especially T cells, NK cells, and B cells. Therefore, the *DOCK8* gene plays a critical role in the survival and function of immune system cells.^[10–12]^

Generally, DOCK8 deficiency is characterized by a combination of immunodeficiencies, including recurrent respiratory or skin infections at a young age. Allergic diseases, malignancies, or autoimmunity could appear as the main features.^[1]^ SLE autoimmunity has been reported in 2 patients.^[3,4]^ The first patient presented with recurrent skin abscesses and sinusitis in early childhood. At the age of 4 years, she had an episode of pneumonia with pneumatocele and was diagnosed with HIES. She developed biopsy-confirmed glomerulonephritis and revealed a positive ANA, indicating the presence of SLE. The second patient with eczema from the age of 8 months was diagnosed with HIES at the age of 33 months through tests performed because of frequent infections. She also had chronic cutaneous lupus, ANA positivity, and antiphospholipid antibody positivity. In our case, the patient had a history of atopic dermatitis without any severe infections until 16 months of age, when he presented with SLE as the first symptom of DOCK8 deficiency. Palpable purpura, oral ulcers, leukopenia, thrombocytopenia, high titer fluorescent ANA with a homogenous pattern, anti-ds DNA positivity, and low serum complement in his evaluation met the “2015 ACR/SLICC Revised Criteria for Diagnosis of Systemic Lupus Erythematosus.”

In DOCK8-deficient patients, the number and the suppressive activity of Tregs, which are responsible for immune tolerance, are decreased. These defective Tregs increase the peripheral B cell autoreactivity and enhance the ability of B cells to produce autoantibodies to self-antigens.^[6]^ There are several mechanisms by which DOCK8 deficiency may impact Treg cell function. It has been suggested that Tregs defect in DOCK8 deficiency occurs through the breakdown of their interaction between the Wiskott–Aldrich protein (WASP) and WASP-interactin protein (WIP), or the activation of STAT3.^[13,14]^ Recently, it has been shown that DOCK8 regulates the suppressive activity of Treg cells through interleukin (IL)-2 in STAT5 signaling.^[15]^ As observed in DOCK8 deficiency, defective Treg cells play a role in the pathogenesis of SLE. Treg defects in SLE are also associated with IL-2 deficiency, as well as with dysregulation of tumor necrosis factor-alpha (TNF-α), major histocompatibility class II and III, and complement components.^[16]^

Since the discovery of the DOCK8 mutation in 2009 as one of the causes of the autosomal recessive form of HIES, genetic evaluation has been conducted to confirm the cause of HIES, and novel mutations in DOCK8 have been reported. Our patient's genetic analysis through gene sequencing identified c.5536A>G in exon 43. This sequence variant, c.5536A>G, predicts an amino acid change of lysine to glutamine at codon 1846 of the DOCK8 protein (p.Lys1846Glu). This sequence variant is of unknown significance, as it has not yet been shown that this mutation alters DOCK8 protein expression. However, this variant was classified as “likely pathogenic,” which provides enough evidence that physicians can use the information in clinical decision making when combined with the presence of other evidence of the disease.^[8]^

Allogeneic HSCT is a fundamental treatment of autoimmune disease and primary immune deficiency, and it has been reported as curative in DOCK8 deficiency.^[17]^ HSCT should be considered as early as possible to prevent life-threatening infections, nonreversible parenchymal organ damage, and complications related to autoimmune disease therapy. Reports of the successful treatment of DOCK8 deficiency through HSCT from human leukocyte antigen (HLA)-matched siblings or unrelated donors have been published.^[18–20]^ More recently, HSCT from haploidentical related donors using post-transplantation cyclophosphamide has been found to be effective in patients with DOCK8 deficiency who lack a fully HLA-matched donor. Ghosh et al^[21]^ and Shah et al^[22]^ reported success in haploidentical HSCT using both Treg depletion and post-transplant immunosuppressants for preventing GvHD. Our patient underwent haploidentical HSCT successfully using a non-myeloablative conditioning regimen without post-transplantation immunosuppressants. Although HSCT cures infection susceptibility and eczematous dermatitis,^[23]^ it remains undetermined whether HSCT cures the autoimmune complication in DOCK8 deficiency. However, our patient has lived for 1 year after HSCT without any recurrence of his autoimmunity. It is necessary to confirm the usefulness of haploidentical HSCT in DOCK8 patients and the curability of autoimmunity via continuous follow-up evaluations after HSCT from all types of donors.

## 4 Conclusion

DOCK8 deficiency can be presented as autoimmune disease such as SLE. When a child is diagnosed with an unusual early-onset SLE, pediatricians should suspect other immunodeficiency diseases. Besides, HSCT can be considered as an attractive and promising option for the treatment of DOCK8 deficiency with autoimmune features.

## Author contributions

All authors have made substantial contributions to the conception of the work. ES, JHL, YSP, HJI, and JL are the treating physicians. BHL conducted the genetic investigation and provided the treatment recommendations. All authors were involved in drafting the work, reviewing, and revising the manuscript. All authors approved the final manuscript as submitted and agree to be accountable for all aspects of the work.

**Conceptualization:** Euri Seo, Beom Hee Lee, Joo Hoon Lee, Young Seo Park, Ho Joon Im, Jina Lee.

**Writing – original draft:** Euri Seo, Jina Lee.

**Writing – review & editing:** Euri Seo, Beom Hee Lee, Joo Hoon Lee, Young Seo Park, Ho Joon Im, Jina Lee.

## References

[1] Biggs CM Keles S Chatila TA. DOCK8 deficiency: insights into pathophysiology, clinical features and management. Clin Immunol 2017;181:75–8. 28625885 10.1016/j.clim.2017.06.003 PMC5555255

[2] Aydin SE Kilic SS Aytekin C. DOCK8 deficiency: clinical and immunological phenotype and treatment options - a review of 136 patients. J Clin Immunol 2015;35:189–98. 25627830 10.1007/s10875-014-0126-0

[3] Yamazaki-Nakashimada M Zaltzman-Girshevich S Garcia de la Puente S. Hyper-IgE syndrome and autoimmunity in Mexican children. Pediatr Nephrol 2006;21:1200–5. 16791602 10.1007/s00467-006-0178-3

[4] Jouhadi Z Khadir K Ailal F. Ten-year follow-up of a DOCK8-deficient child with features of systemic lupus erythematosus. Pediatrics 2014;134:1458–63. 10.1542/peds.2013-1383 25332498

[5] AlKhater SA. CNS vasculitis and stroke as a complication of DOCK8 deficiency: a case report. BMC Neurol 2016;16:54. 27113444 10.1186/s12883-016-0578-3 PMC4845487

[6] Janssen E Morbach H Ullas S. Dedicator of cytokinesis 8-deficient patients have a breakdown in peripheral B-cell tolerance and defective regulatory T cells. J Allergy Clin Immunol 2014;134:1365–74. 25218284 10.1016/j.jaci.2014.07.042 PMC4261031

[7] Ohl K Tenbrock K. Regulatory T-cells in systemic lupus erythematosus. IL-2 is decisive for loss of tolerance. Z Rheumatol 2016;75:253–64. 26975190 10.1007/s00393-016-0060-z

[8] Richards S Aziz N Bale S. Standards and guidelines for the interpretation of sequence variants: a joint consensus recommendation of the American College of Medical Genetics and Genomics and the Association for Molecular Pathology. Genet Med 2015;17:405–24. 25741868 10.1038/gim.2015.30 PMC4544753

[9] Ruusala A Aspenström P. Isolation and characterisation of DOCK8, a member of the DOCK180-related regulators of cell morphology. FEBS Lett 2004;572:159–66. 15304341 10.1016/j.febslet.2004.06.095

[10] Crawford G Enders A Gileadi U. DOCK8 is critical for the survival and function of NKT cells. Blood 2013;122:2052–61. 23929855 10.1182/blood-2013-02-482331 PMC3778549

[11] Jabara HH McDonald DR Janssen E. DOCK8 functions as an adaptor that links TLR-MyD88 signaling to B cell activation. Nat Immunol 2012;13:612–20. 22581261 10.1038/ni.2305 PMC3362684

[12] Lambe T Crawford G Johnson AL. DOCK8 is essential for T-cell survival and the maintenance of CD8+ T-cell memory. Eur J Immunol 2011;41:3423–35. 21969276 10.1002/eji.201141759 PMC3517112

[13] Marangoni F Trifari S Scaramuzza S. WASP regulates suppressor activity of human and murine CD4(+)CD25(+)FOXP3(+) natural regulatory T cells. J Exp Med 2007;204:369–80. 17296785 10.1084/jem.20061334 PMC2118740

[14] Keles S Charbonnier LM Kabaleeswaran V. Dedicator of cytokinesis 8 regulates signal transducer and activator of transcription 3 activation and promotes TH17 cell differentiation. J Allergy Clin Immunol 2016;138:1384–94. 27350570 10.1016/j.jaci.2016.04.023 PMC5099100

[15] Singh AK Eken A Hagin D. DOCK8 regulates fitness and function of regulatory T cells through modulation of IL-2 signaling. JCI Insight 2017;2:e94275. 10.1172/jci.insight.94275 PMC5841873 28978795

[16] von Spee-Mayer C Siegert E Abdirama D. Low-dose interleukin-2 selectively corrects regulatory T cell defects in patients with systemic lupus erythematosus. Ann Rheum Dis 2016;75:1407–15. 26324847 10.1136/annrheumdis-2015-207776

[17] Tyndall A. Successes and failures of stem cell transplantation in autoimmune diseases. Hematology Am Soc Hematol Educ Program 2011;280–4. 22160046 10.1182/asheducation-2011.1.280

[18] Kuşkonmaz B Ayvaz D Tezcan İ. Successful hematopoietic stem cell transplantation after myeloablative conditioning in three patients with dedicator of cytokinesis 8 deficiency (DOCK8) related Hyper IgE syndrome. Bone Marrow Transplant 2018;53:339–43. 29269803 10.1038/s41409-017-0040-1

[19] Ghosh S Schuster FR Fuchs I. Treosulfan-based conditioning in DOCK8 deficiency: complete lympho-hematopoietic reconstitution with minimal toxicity. Clin Immunol 2012;145:259–61. 23128504 10.1016/j.clim.2012.10.003

[20] Cuellar-Rodriguez J Freeman AF Grossman J. Matched related and unrelated donor hematopoietic stem cell transplantation for DOCK8 deficiency. Biol Blood Marrow Transplant 2015;21:1037–45. 25636378 10.1016/j.bbmt.2015.01.022 PMC4426076

[21] Ghosh S Schuster FR Adams O. Haploidentical stem cell transplantation in DOCK8 deficiency: successful control of pre-existing severe viremia with a TCRaß/CD19-depleted graft and antiviral treatment. Clin Immunol 2014;152:111–4. 24667686 10.1016/j.clim.2014.03.006

[22] Shah NN Freeman AF Su H. Haploidentical related donor hematopoietic stem cell transplantation for dedicator-of-cytokinesis 8 deficiency using post-transplantation cyclophosphamide. Biol Blood Marrow Transplant 2017;23:980–90. 28288951 10.1016/j.bbmt.2017.03.016 PMC5757872

[23] Al-Herz W Chu JI van der Spek J. Hematopoietic stem cell transplantation outcomes for 11 patients with dedicator of cytokinesis 8 deficiency. J Allergy Clin Immunol 2016;138:852–9. 27130861 10.1016/j.jaci.2016.02.022 PMC5354354

